# Unbiased evaluation of rapamycin's specificity as an mTOR inhibitor

**DOI:** 10.1111/acel.13888

**Published:** 2023-05-24

**Authors:** Filippo Artoni, Nina Grützmacher, Constantinos Demetriades

**Affiliations:** ^1^ Max Planck Institute for Biology of Ageing (MPI‐AGE) Cologne Germany; ^2^ Cologne Graduate School of Ageing Research (CGA) Cologne Germany; ^3^ Cologne Excellence Cluster on Cellular Stress Responses in Aging‐Associated Diseases (CECAD) University of Cologne Cologne Germany

**Keywords:** ageing, mTORC1, proteomics, rapamycin, sirolimus

## Abstract

Rapamycin is a macrolide antibiotic that functions as an immunosuppressive and anti‐cancer agent, and displays robust anti‐ageing effects in multiple organisms including humans. Importantly, rapamycin analogues (rapalogs) are of clinical importance against certain cancer types and neurodevelopmental diseases. Although rapamycin is widely perceived as an allosteric inhibitor of mTOR (mechanistic target of rapamycin), the master regulator of cellular and organismal physiology, its specificity has not been thoroughly evaluated so far. In fact, previous studies in cells and in mice hinted that rapamycin may be also acting independently from mTOR to influence various cellular processes. Here, we generated a gene‐edited cell line that expresses a rapamycin‐resistant mTOR mutant (mTOR^RR^) and assessed the effects of rapamycin treatment on the transcriptome and proteome of control or mTOR^RR^‐expressing cells. Our data reveal a striking specificity of rapamycin towards mTOR, demonstrated by virtually no changes in mRNA or protein levels in rapamycin‐treated mTOR^RR^ cells, even following prolonged drug treatment. Overall, this study provides the first unbiased and conclusive assessment of rapamycin's specificity, with potential implications for ageing research and human therapeutics.

## Graphical abstract

Although rapamycin is widely used as an allosteric inhibitor of mTOR, its specificity and selectivity has not been thoroughly evaluated before. Here, by performing unbiased transcriptomics and proteomics in a gene‐edited cell line that expresses a rapamycin‐resistant mTOR mutant, Artoni et al. reveal the striking specificity of this compound towards mTOR in cells.

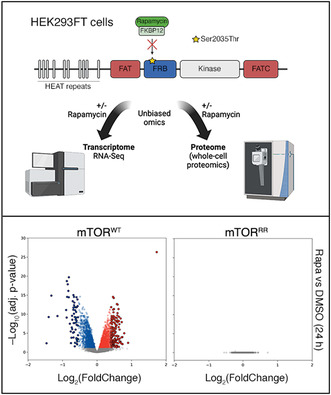

**Abbreviations**
Cas9: CRISPR‐associated protein 9
CRISPR: Clustered Regularly Interspaced Short Palindromic Repeats
EMA: European Medicine Agency
FDA: Federal Food and Drug Administration
TGFβ: Transforming Growth Factor beta
TRP channel: transient receptor potential channel

## 1 INTRODUCTION

Rapamycin (also known as sirolimus) is a naturally occurring macrolide compound which was originally isolated from soil bacteria on Easter Island (Rapa Nui) in 1972 (Arriola Apelo & Lamming, 2016; Benjamin et al., 2011; Li et al., 2014). Rapamycin was primarily used in the clinic as an anti‐fungal agent until 1999 when it was approved by the FDA for the prevention of kidney transplant rejection and later for the treatment of advanced kidney cell carcinoma. Its immunosuppressive and anti‐proliferative properties are thought to be largely mediated by inhibition of mTOR, a serine/threonine kinase that functions as the master regulator of most cellular functions, including immune cell activation and cell growth control (Arriola Apelo & Lamming, 2016; Benjamin et al., 2011; Fernandes & Demetriades, 2021; Li et al., 2014). At the molecular level, rapamycin inhibits mTORC1 (mTOR complex 1) activity as a complex with the cytosolic immunophilin FKBP12 (FK506‐binding protein 12). Binding of this drug‐protein complex to mTOR blocks access to its catalytic site and prevents the phosphorylation of key substrates like S6K (ribosomal protein S6 kinase) (Chung et al., 1992). Additional immunophilins such as FKBP12.6, FKBP51, and FKBP52 have also been reported to bind and shape the pharmacology of rapamycin (März et al., 2013).

Over the last two decades, rapamycin has gained renewed interest after multiple studies uncovered its powerful anti‐ageing properties (Arriola Apelo & Lamming, 2016; Benjamin et al., 2011; Fernandes & Demetriades, 2021; Li et al., 2014). Rapamycin has been repeatedly shown to extend lifespan and/or healthspan in worms (Robida‐Stubbs et al., 2012), flies (Bjedov et al., 2010; Castillo‐Quan et al., 2019; Schinaman et al., 2019), and mice (Bitto et al., 2016; Fok et al., 2014; Harrison et al., 2009). It extends chronological age in yeast (Powers et al., 2006) and reduces markers of senescence in cultured cells (Wang et al., 2017). More recently, studies conducted on marmoset monkeys have demonstrated rapamycin to have a good safety and tolerability profile (Lelegren et al., 2016; Tardif et al., 2015) thus paving the way for human trials. Currently, trials are underway both on companion dogs (Creevy et al., 2022) and humans (NCT04488601) with preliminary evidence suggesting improvement in cardiac function in middle‐aged dogs who received rapamycin for 10 weeks (Urfer et al., 2017). Furthermore, RAD001/everolimus, a rapamycin analogue, was shown to improve immune function and the response to influenza vaccination in elderly humans (Kennedy & Pennypacker, 2015; Mannick et al., 2014). Notably, rapamycin extends lifespan at doses much lower than the ones used to achieve immunosuppression in transplant patients thus minimizing potential side effects. Finally, even transient, intermittent, or late‐life rapamycin administration has been shown to extend lifespan in flies and mice thus making rapamycin an attractive option for human use as an anti‐ageing compound (Bitto et al., 2016; Harrison et al., 2009; Partridge et al., 2020).

The target specificity of rapamycin has recently been debated. On one hand, *in vitro* kinase activity assays showed other serine/threonine kinases, besides mTOR, to be largely insensitive to rapamycin up to concentrations at the micromolar range. Likewise, receptor‐binding assays indicated that many ligand‐receptor interactions remain unaffected by rapamycin, with the possible exception of histamine I binding (EMA, 2005). On the other hand, accumulating evidence in the literature suggest that rapamycin may exert some of its effects via mTOR‐independent mechanisms. This is also supported by the fact that most kinase inhibitors demonstrate very low target selectivity (Hantschel, 2015; Hantschel et al., 2012; Karaman et al., 2008). For instance, rapamycin was suggested to block the exercise‐induced accumulation of ribosomal RNA (rRNA) in the skeletal muscle of both wild‐type mice and mice containing a rapamycin‐resistant mTOR allele (Goodman et al., 2011). Moreover, rapamycin and other rapalogs were shown to directly bind and activate the lysosomal mucolipin TRP channel (TRPML1; also known as MCOLN1) independently of mTOR inhibition (Zhang et al., 2019). Finally, since FKBPs serve as chaperones for proper folding of several proteins (Bonner & Boulianne, 2017; Bultynck et al., 2001; Galfre et al., 2012; Vervliet et al., 2015; Wang et al., 1996), it can be speculated that their binding to rapamycin may be influencing cellular physiology via altering the interaction of FKBPs to their client proteins.

Here, to conclusively address this important unresolved issue, we generated a rapamycin‐resistant cell line by editing a single base in the *MTOR* gene at its endogenous locus (Choi et al., 1996; Hosoi et al., 1999; Lorenz & Heitman, 1995) and assessed how rapamycin affects the cellular transcriptome and proteome in an unbiased manner. These experiments unraveled an impressive specificity of rapamycin towards mTOR, with the rapamycin effects on gene expression and protein levels being virtually non‐existent in the mTOR‐mutant cells.

## 2 RESULTS

### 2.1 Generation and characterization of a gene‐edited cell line that expresses rapamycin‐resistant mTOR

**FIGURE 1:**
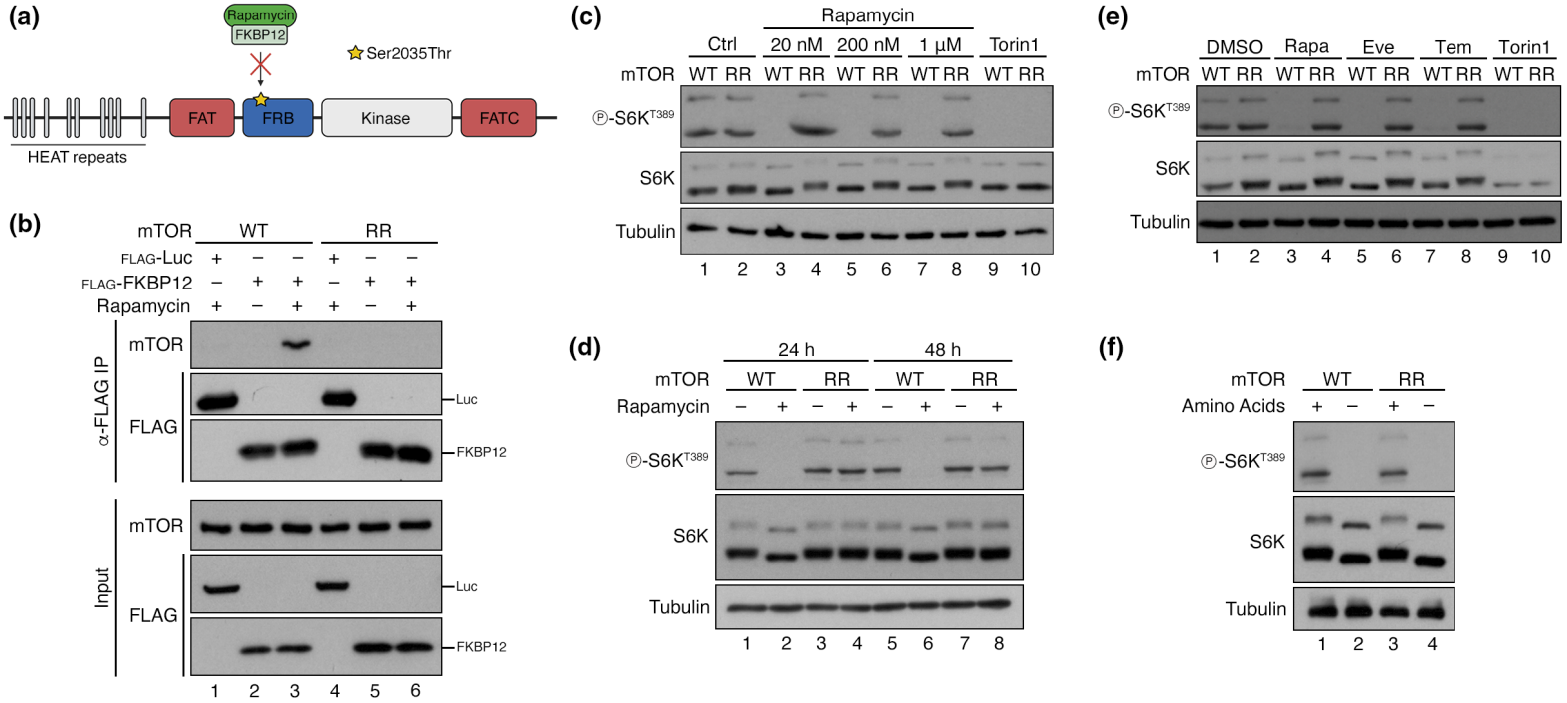
Generation and characterization of a gene‐edited cell line that expresses rapamycin‐resistant mTOR. (a) Schematic model of the mTOR protein depicting key domains and the location of the Ser2035Thr substitution in the FRB domain that prevents its inhibition by rapamycin/FKBP12. (b) Diminished binding of FKBP12 to the mTOR^RR^ mutant protein. Co‐immunoprecipitation experiments in control (WT) or mTOR^RR^ HEK293FT cells, transiently expressing FLAG‐tagged FKBP12 or Luciferase (Luc) as negative control, treated with rapamycin (20 nM, 1 h) or DMSO. Binding of mTOR to FKBP12 was analysed by immunoblotting as indicated. (c) mTOR^RR^ does not respond to rapamycin even at extremely high concentrations. Immunoblots with lysates from HEK293FT WT and mTOR^RR^ cells treated for 1 h with 20 nM to 1 μM rapamycin as indicated, 250 nM Torin1, or DMSO as control. mTORC1 activity was assessed by S6K phosphorylation. (d) mTOR^RR^ is resistant to long‐term rapamycin treatment. Immunoblots with lysates from HEK293FT WT and mTOR^RR^ cells treated with DMSO or rapamycin (20 nM) for 24 or 48 h, probed with the indicated antibodies. (e) mTOR^RR^ shows resistance to multiple rapalogs. Immunoblots with lysates from HEK293FT WT and mTOR^RR^ cells treated with DMSO, rapamycin (Rapa; 20 nM), everolimus (Eve; 20 nM), temsirolimus (Tem; 20 nM), or Torin1 (250 nM) for 1 h, probed with the indicated antibodies. (f) Cell expressing mTOR^RR^ have active mTORC1 that responds properly to AA starvation. Immunoblots with lysates from HEK293FT WT and mTOR^RR^ cells treated with control (+) or AA‐free media (–) for 1 h, probed with the indicated antibodies.

Mutations in the mTOR FRB (FKBP‐rapamycin‐binding) domain that disrupt its interaction with FKBP12 and confer rapamycin resistance to mTOR have been described almost 30 years ago (Brown et al., 1995; Chen et al., 1995; Choi et al., 1996; Hara et al., 1997; Hosoi et al., 1999; Lorenz & Heitman, 1995). Although such mTOR mutants have been used in previous studies, usually expressed exogenously in cells or via transgenic expression in mouse tissues, the presence of endogenous wild‐type mTOR has complicated the interpretation of these results (Ge et al., 2009; Goodman et al., 2011; Luo et al., 2015; Zhang et al., 2000). Moreover, a thorough analysis of rapamycin's effects in such cellular models, beyond single readouts, is lacking. Therefore, to probe whether rapamycin also acts through mTOR‐independent mechanisms or exclusively via mTOR inhibition, we used CRISPR/Cas9‐mediated gene editing to generate a HEK293FT cell line that expresses a Ser2035Thr mTOR mutant (Figure 1a, Figure S1) that was previously described to be rapamycin‐resistant (Brown et al., 1995; Chen et al., 1995; Choi et al., 1996; Hara et al., 1997; Hosoi et al., 1999; Lorenz & Heitman, 1995). Intuitively, we named this mutant and the associated cell line, mTOR^RR^ (mTOR rapamycin‐resistant). Importantly, as we mutated *MTOR* in the endogenous locus, no wild‐type mTOR is expressed in these cells, verified by genomic DNA sequencing (Figure S1). Consistent with previous reports, the rapamycin‐induced interaction of FLAG‐tagged FKBP12 with wild‐type mTOR, was completely abrogated in mTOR^RR^ (Figure 1b). Of note, the catalytic activity of mTORC1 containing mTOR^RR^, as assessed by the phosphorylation of its direct substrate S6K, was indistinguishable from that in control cells and was diminished by treatment with Torin1, an ATP‐competitive mTOR inhibitor (Figure 1c). In contrast, mTOR^RR^ demonstrated complete resistance to rapamycin even when cells were treated with this compound at micromolar concentrations (Figure 1c), or when the treatment was extended to 24 or 48 h (Figure 1d). Similar to rapamycin, mTOR^RR^ also exhibited full resistance to other rapalogs like everolimus and temsirolimus (Figure 1e), but responded properly to amino acid (AA) starvation, showing that the regulation of mTORC1 by other inhibitory stimuli is unperturbed in mTOR^RR^ cells (Figure 1f). In sum, the mTOR^RR^ HEK293FT cell line is a ‘clean’, reliable and robust model to investigate rapamycin's specificity towards mTOR.

### 2.2 Rapamycin alters gene expression exclusively via mTOR inhibition

**FIGURE 2:**
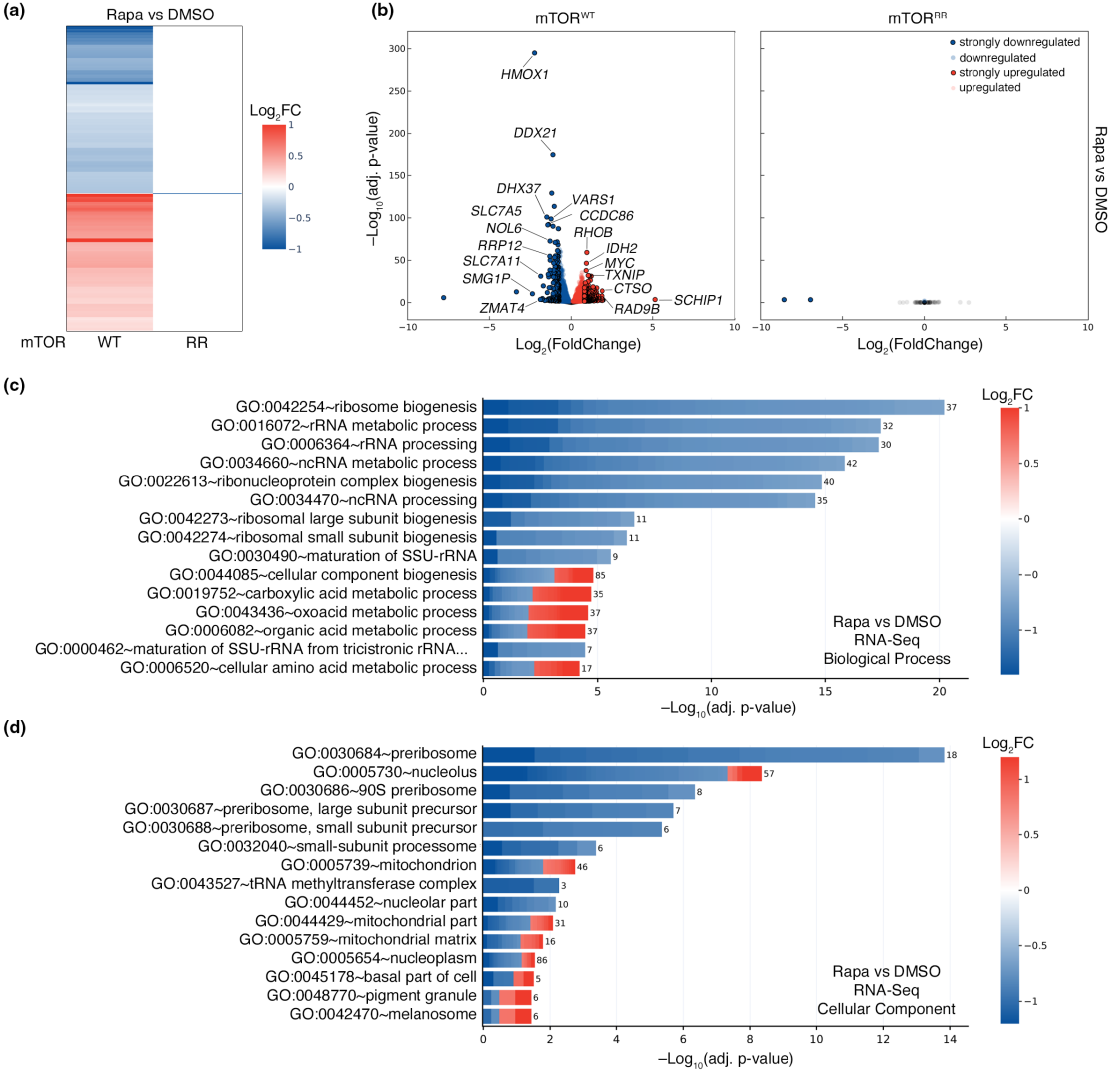
Rapamycin alters gene expression exclusively via mTOR inhibition. (a) Heatmap depicting the rapamycin‐induced changes in gene expression in HEK293FT WT and mTOR^RR^ cells. RNA‐seq data from cells treated with rapamycin (20 nM, 24 h) expressed as log‐transformed fold change (Log_2_FC). Only statistically‐significant changes (adj. *p*‐value <0.05) are shown. (b) Volcano plots showing the rapamycin‐induced changes in gene expression in HEK293FT WT (left) and mTOR^RR^ (right) cells from the RNA‐seq experiment described in (a). Genes that are significantly (adj. *p*‐value <0.05) down‐ or upregulated by rapamycin are shown in blue or red respectively. Strongly down‐ (Log_2_FC < –0.75) or upregulated (Log_2_FC > +0.8) genes are shown with black outline. Unaffected genes (adj. *p*‐value >0.05) shown as grey dots. Selected genes are marked in the plot. (c) Biological process (BP) GO term analysis using the genes that are strongly down‐ (blue) or upregulated (red) by rapamycin in WT cells as described in (b). The colour of each box in the cell plot represents log‐transformed fold change values for each gene in rapamycin‐ vs DMSO‐treated cells. The number of genes in the selected dataset for each GO term is shown on the right side of each bar. (d) As in (c), but for Cellular Component (CC) GO term analysis.

Having validated our experimental model, we then treated control (WT) and mTOR^RR^ cells with rapamycin for 24 h and performed RNA‐seq experiments to investigate its effects on global gene expression. In line with mTOR—directly or indirectly—regulating the activity of several transcription factors (Hardwick et al., 1999; Laplante & Sabatini, 2013), we detected more than 5000 genes whose expression changed significantly in WT cells upon rapamycin treatment (Figure 2a,b, Table S1). Our analysis identified several genes that are known to be affected by mTOR inhibition (e.g., HMOX1, RHOB, MYC) (Bayeva et al., 2012; Gordon et al., 2015; Jin et al., 2013; Sun et al., 2022; Visner et al., 2003) (Figure 2b) thus validating our experimental setup. Similarly, rapamycin decreased the expression of AA transporters like SLC7A5 and SLC7A11, which are known to be regulated downstream of an mTORC1‐ATF4 axis (Torrence et al., 2021) (Figure 2b, Tables S1 and S2).

Gene ontology (GO) term enrichment analysis, using the differentially regulated genes that are strongly down‐ or upregulated by rapamycin (Log_2_FC < −0.75 and Log_2_FC > +0.8, respectively; and adjusted *p*‐value <0.05), revealed a strong enrichment of ribosome‐related Biological Process (BP) and Cellular Component (CC) terms (e.g., BP:GO:0042254 ~ ribosome biogenesis; CC:GO:0030684 ~ preribosome) (Figure 2c,d, Table S2). Confirming the well‐known role of mTOR in promoting ribosome biogenesis and positively regulating rRNA expression (Mayer & Grummt, 2006; Powers & Walter, 1999), all genes that fall under these terms (e.g., RRP12, RRP9, RRS1, RRP1, RPF2, NOP16, NOL6, DDX21, MRTO4, MRM1) were found to be downregulated by rapamycin (Figure 2c,d, Table S2). Interestingly, we also observed a strong enrichment of terms related to mitochondria‐resident proteins (e.g., CC:GO:0005739 ~ mitochondrion; CC:GO:0005759 ~ mitochondrial matrix) and associated mitochondrial functions (e.g., BP:GO:0019752 ~ carboxylic acid metabolic process) among the genes that are differentially regulated by rapamycin (Figure 2c,d, Table S2). Similar results were obtained when performing the GO analysis with more relaxed criteria including all significantly down‐ and upregulated genes, instead of setting cut‐offs for those that change robustly upon rapamycin (Figure S2a,b, Table S3). Strikingly, unlike the pervasive transcriptional effects of rapamycin in control cells, we found only two genes (*UPK3BL1*, *AC007326.4*) whose expression was altered in mTOR^RR^ cells treated with rapamycin, based on the same selection criteria (Figure 2a,b, Table S1). These data indicate that rapamycin practically regulates transcription exclusively via its direct inhibitory effect on mTOR.

### 2.3 Rapamycin alters the cellular proteome exclusively through mTOR

In addition to their involvement in transcriptional regulation, the best‐described role of rapamycin and mTORC1 is in the control of *de novo* protein synthesis via regulating the phosphorylation and activity of—direct or indirect—mTORC1 targets like S6K, 4E‐BP1, and S6, with only certain 4E‐BP1 phospho‐sites being rapamycin‐sensitive (Thoreen et al., 2009, 2012). Therefore, we next sought to investigate the rapamycin‐induced changes in the cellular proteome and explore the extent to which this happens because of mTOR inhibition.

**FIGURE 3:**
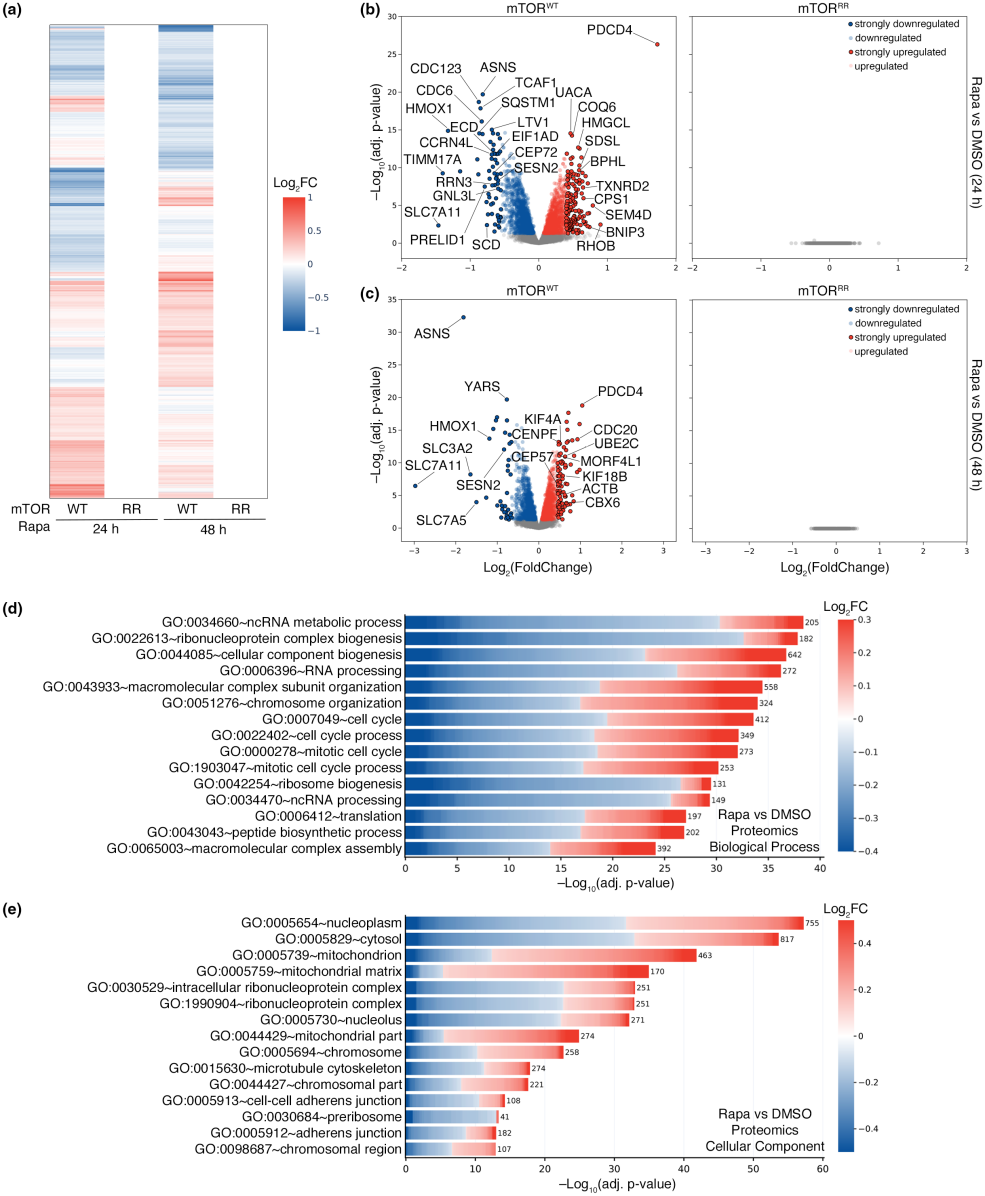
Rapamycin alters the cellular proteome exclusively via mTOR inhibition. (a) Heatmap depicting the rapamycin‐induced changes in the proteome of HEK293FT WT and mTOR^RR^ cells. Shown are whole‐proteome quantitative mass‐spectrometry data from cells treated with rapamycin (20 nM) for 24 or 48 h expressed as log‐transformed fold change (Log_2_FC). Only statistically‐significant changes (adj. *p*‐value <0.05) are shown. (b) Volcano plots showing the rapamycin‐induced proteomic changes in HEK293FT WT (left) and mTOR^RR^ (right) cells from the mass‐spectrometry experiment described in (a). Proteins that are significantly (adj. *p*‐value <0.05) down‐ or upregulated by rapamycin (20 nM, 24 h) are shown in blue or red respectively. Most strongly down‐ (Log_2_FC <–0.55) or upregulated (Log_2_FC > +0.4) proteins are shown with black outline. Unaffected proteins (adj. *p*‐value >0.05) shown as grey dots. Selected proteins are marked in the plot. (c) As in (b), but for cells treated with 20 nM rapamycin (or DMSO) for 48 h. Strongly down‐ (Log_2_FC <–0.65) or upregulated (Log_2_FC >+0.45) proteins are shown with black outline. (d) Biological process (BP) GO term analysis using the proteins that are significantly down‐ (blue) or upregulated (red) by 24 h rapamycin in WT cells as described in (b). The color of each box in the cell plot represents log‐transformed fold change values for each protein in rapamycin‐ vs DMSO‐treated cells. The number of proteins in the selected dataset for each GO term is shown on the right side of each bar. (e) As in (d), but for Cellular Component (CC) GO term analysis.

To this end, we treated control and mTOR^RR^ cells with rapamycin for 24 or 48 h and performed whole‐proteome quantitative mass spectrometry experiments. Out of a total of approximately 7500 proteins that were detected and quantified, the levels of more than 2500 and 2300 proteins changed significantly in WT cells treated with rapamycin for 24 or 48 h, respectively (Figure 3a–c, Table S4). Similar to what we observed in transcriptomic analyses, these hits include multiple proteins belonging to pathways that have been previously described to be regulated by rapamycin and mTOR (e.g., PDCD4, RHOB, HMOX1, SQSTM1, PRELID1, SCD, SESN2) (Bayeva et al., 2012; Dorrello et al., 2006; Gordon et al., 2015; Jin et al., 2013; Ko et al., 2017; Mauvoisin et al., 2007; Sun et al., 2022; Visner et al., 2003; Wall et al., 2008; Zhu et al., 2020) as well as several amino acid transporters (SLC7A11, SLC38A10, SLC3A2, SLC7A5) (Graber et al., 2017; Nachef et al., 2021; Torrence et al., 2021; Zhang et al., 2021) (Figure 3b,c, Table S4). Remarkably, however, none of the 7574 detected proteins were differentially regulated in rapamycin‐treated mTOR^RR^ cells, even after prolonged drug treatment (Figure 3a–c, Table S4), again showing complete absence of mTOR‐independent effects of rapamycin.

Consistent with our observations from RNA‐seq experiments, GO analysis using all proteins that are significantly down‐ or upregulated (adj. *p*‐value <0.05) upon 24‐h rapamycin treatment in WT cells showed strong enrichment of terms related to ribosomes and translation (e.g., BP:GO:0022613 ~ ribonucleoprotein complex biogenesis; BP:GO:0042254 ~ ribosome biogenesis; BP:GO:0006412 ~ translation) (Figure 3d, Table S5) with the majority of proteins that belong to this category being downregulated (e.g., CDC123, LTV1, RRN3, EIF1AD, SESN2, GNL3L, CCDC86, WDR74, NUFIP1, CDK4) (Figure 3b,d, Tables S4 and S5). Another prominent group of GO terms that is enriched in the proteomic analysis includes terms related to the cell cycle (e.g., BP:GO:0007049 ~ cell cycle, BP:GO:0000278 ~ mitotic cell cycle) (Figure 3d, Tables S4 and S5) with proteins being either down‐ or upregulated upon rapamycin treatment (e.g., CDC123, CDC6, ASNS, ECD, CEP72, PDCD4, RHOB, DHFR, TRIOBP, H1F0) (Figure 3d). Of note, this is in line with the well‐established role of rapamycin and mTOR in the regulation of cell proliferation (Dowling et al., 2010). Similar results were obtained when analyzing the respective dataset (all significantly‐regulated proteins; adj. *p*‐value <0.05) from 48‐h rapamycin treatment in WT cells (Figure 3a,c, Figure S3, Table S6).

Furthermore, in line with the robust expression changes in genes related to mitochondria (Figure 2c,d), we observed similar effects in the levels of mitochondrial proteins, as shown by the strong enrichment of related GO terms (e.g., CC:GO:0005739 ~ mitochondrion; CC:GO:0005759 ~ mitochondrial matrix) (Figure 3e, Tables S4 and S5). Interestingly, when performing the GO term analysis using only the strongly up‐ or downregulated proteins, the enrichment of terms related to mitochondrial proteins became even more prominent (Figure S4, Table S7) with most mitochondria‐related proteins being upregulated by rapamycin (e.g., BNIP3, BNIP3L, CPS1, TXNRD2, SDSL, ACSF2, BPHL, HMGCL, HSD17B8, SFN) (Figure S4, Tables S4 and S7).

Finally, although a connection between mTOR activity and cell adhesion has been described before, the underlying mechanisms are less clear (Asrani et al., 2017; Chen et al., 2015). Interestingly, our proteomic analysis showed that rapamycin treatment led to significant changes in the levels of a large number of proteins that are related to adherens and anchoring junctions (CC:GO:0005912 ~ adherens junction; CC:GO:0070161 ~ anchoring junction), most of which are downregulated under these conditions (e.g., GJA1, SLC3A2, RANGAP1, DSP, FASN, APC, EEF2, TNKS1BP1, EHD4, CNN3) (Figure 3b,e, Tables S4 and S7). This suggests that some of the effects of rapamycin/mTOR on cell adhesion may stem from changes in the junctional proteome.

In sum, our unbiased interrogation of rapamycin's effects in cells reveals an extraordinary specificity of this compound towards mTOR, and—at the same time—provides a thorough evaluation of its role on the cellular transcriptome and proteome.

## 3 DISCUSSION

According to previous studies, the majority of inhibitory compounds that are used in research or in therapeutics demonstrate off‐target effects, influencing the activity of additional signaling molecules that can also be structurally‐unrelated to their presumed targets. Sometimes, inhibitors even show higher potency towards off‐targets compared to on‐target effects (Bain et al., 2003, 2007; Davies et al., 2000; Davis et al., 2011; Fedorov et al., 2007). In fact, very few FDA‐approved kinase inhibitors have demonstrated high target selectivity, whereas the majority influences the activity of 10–100 off‐target kinases (Hantschel, 2015; Hantschel et al., 2012; Karaman et al., 2008). Importantly, the low specificity and selectivity of most kinase inhibitors greatly complicates the interpretation of data originating from their use and has important implications for their applicability in both research and therapeutics.

Surprisingly, although rapamycin has been used as an mTOR inhibitor for more than three decades, its specificity towards this key signaling hub has not been thoroughly evaluated so far. In fact, previous studies have hinted at the existence of mTOR‐independent functions of rapamycin in cells and in transgenic mouse models, thus underscoring the need for a detailed evaluation of its specificity. In transgenic mice overexpressing a rapamycin‐resistant mTOR mutant in skeletal muscles, rapamycin was still able to partially suppress mechanical‐loading‐induced ribosome biogenesis (Goodman et al., 2011). It is worth noting however, that these transgenic mice were maintained as hemizygotes with the mTOR^RR^ allele expressed on top of endogenous wild‐type mTOR (Ge et al., 2009; Goodman et al., 2011), which does not allow for definitive conclusions to be drawn from such experiments. Indeed, the observed rapamycin effects that were previously interpreted as mTOR‐independent can also be ascribed to the inhibition of endogenous wild‐type mTOR molecules. This is also supported by our findings, using a gene‐edited cell line that expresses rapamycin‐resistant mTOR from the endogenous *MTOR* locus, while lacking expression of wild‐type mTOR: unlike the previously described mTOR^RR^ transgenic mice, our mTOR^RR^ cells are fully resistant to rapamycin, which is demonstrated by unaffected mTORC1 activity, abrogated FKBP12‐mTOR binding, and complete lack of transcriptional or proteomic changes in response to rapamycin treatment.

A more recent study reported that micromolar concentrations of rapamycin (or of other rapalogs like everolimus and temsirolimus) can directly bind to TRPML1/MCOLN1, the primary lysosomal calcium release channel, and enhance its activity in an mTOR‐independent manner (Zhang et al., 2019). Although rapalogs may indeed act on additional substrates at such concentrations, these are two to three orders of magnitude higher than those classically used in cell culture experiments (2–20 nM; like those we used for the transcriptomic and proteomic analyses described here) or—most importantly—those that are detected in the blood of patients that take rapamycin. More specifically, whole‐blood concentrations (WBC) of rapamycin/sirolimus are in the 10–20 nM range in renal transplant patients to prevent graft rejection (Meier‐Kriesche & Kaplan, 2000). In patients treated with everolimus against metastatic renal cell carcinoma, the median WBC is approximately 15 nM (Takasaki et al., 2019), while plasma concentrations of temsirolimus for relapsed/refractory multiple myeloma average around 9 nM (Farag et al., 2009). Higher doses of temsirolimus have only been used as a last resort in clinical trials against aggressive forms of lymphoma with WBC values reaching 500–600 nM (Hudes et al., 2007), which is still more than 25 times lower than the rapamycin concentration required to half‐maximally activate TRPML1 (Zhang et al., 2019). Thus, the rapamycin‐mediated TRPML1 activation, while observed *in vitro*, is not likely to be physiologically relevant for research and therapeutics. Furthermore, rapamycin doses aimed at slowing or reversing ageing are generally even lower than those used for immunosuppression and cancer treatment (Bitto et al., 2016; Bjedov et al., 2010; Castillo‐Quan et al., 2019; Fok et al., 2014; Harrison et al., 2009; Robida‐Stubbs et al., 2012; Schinaman et al., 2019), further suggesting that TRPML1 activation is unlikely to be involved in rapamycin‐mediated lifespan extension.

Rapamycin acts as an allosteric mTOR inhibitor in complex with FKBP12 and other FKBP family members (Chung et al., 1992; Marz et al., 2013). Most FKBPs possess peptidyl‐prolyl isomerase (PPIase) activity, hence functioning as protein folding chaperones for a variety of different proteins (Harrar et al., 2001; Kolos et al., 2018). For instance, FKBP12 and FKBP12.6 have been shown to bind and modulate the activity of ryanodine receptors (RyRs) and inositol 1,4,5‐trisphosphate receptors (IP3Rs) (Bultynck et al., 2001; Galfre et al., 2012; Vervliet et al., 2015), which are channels involved in intracellular calcium release. FKBP12 has also been shown to inhibit TGFβ family type I receptors (Wang et al., 1996). Likewise, the larger FKBP51 and FKBP52 chaperones control glucocorticoid receptor (GR) localization and activity (Fries et al., 2017) as well as the protein levels of Argonaute 2 (AGO2) (Martinez et al., 2013), an essential component of the RNA‐induced silencing complex (RISC). Hence, it would be reasonable to speculate that binding of rapamycin to different FKBP proteins may influence the folding—and thus function—of client proteins beyond mTOR inhibition. Because any changes in receptor or signaling pathway activities, organelle function, metabolism, or other cellular processes are eventually translated to changes in gene or protein expression, we here analyzed the cellular transcriptome and proteome to interrogate the effects of rapamycin treatment in an unbiased manner. These experiments revealed a striking dependence of rapamycin on mTOR inhibition to influence cells, with mTOR^RR^‐expressing cells demonstrating virtually no effects upon treatment with this drug. In sum, although rapamycin could—in theory—affect cells also independently from mTOR inhibition (via TRPML1 or through FKBP‐dependent mechanisms), this is not the case, at least for rapamycin concentrations that are within the nanomolar range used in research or found in the blood of patients treated with this drug or its analogues. One possible reason for this phenomenal specificity and selectivity of rapamycin towards mTOR may be the fact that, unlike ATP‐competitive kinase inhibitors that bind directly to their target, this compound inhibits mTOR activity in complex with FKBP12: only when the drug‐protein holocomplex interacts with the FRB domain of mTOR, it blocks access of its substrates to its catalytic site and prevents their phosphorylation (Chung et al., 1992).

In addition to assessing rapamycin's specificity towards mTOR, we here also investigated how it influences gene expression and whole‐cell protein levels in control cells that express wild‐type mTOR. Consistent with the role of mTORC1 in regulating cap‐dependent translation (Fonseca et al., 2014; Holz et al., 2005; Liu & Sabatini, 2020; Ma & Blenis, 2009; Thoreen et al., 2012) and with rapamycin's ability to repress both global and specific translation of key subsets of transcripts (Dickinson et al., 2011; Huo et al., 2011; Nandagopal & Roux, 2015; Tsukumo et al., 2016; Wang et al., 2007), these transcriptomics and proteomics datasets identified several thousands of genes and proteins whose expression changes in rapamycin‐treated cells. For instance, highlighting the well‐described role of rapamycin/mTOR on ribosome biogenesis, rapamycin treatment strongly downregulated the expression of genes encoding for ribosomal proteins, tRNA synthetases (Kim et al., 2017; Lee et al., 2012) and other accessory proteins involved in this process (Mayer & Grummt, 2006; Powers & Walter, 1999) (Figure 2, Figure S2). Accordingly, we observed a strong enrichment of proteins related to non‐coding RNA metabolic processes and ribonucleoprotein complex biogenesis among those downregulated by rapamycin (Figure 3, Figure S3). Furthermore, both our RNA‐seq and proteomics data also confirm previous studies about rapamycin's role in regulating mitochondrial function (Morita et al., 2017; Ramanathan & Schreiber, 2009; Rosario et al., 2019; Schieke et al., 2006; Villa‐Cuesta et al., 2014) (Figures 2 and 3, Figures S2 and S3). Interestingly, we find that proteins that are associated with adherens/anchoring junctions are enriched among those downregulated by rapamycin, which may suggest a role for mTOR in the regulation of cell–cell and cell‐matrix contacts (Figure 3e and Figure S3b). Finally, we observe an enrichment of terms associated with the cell cycle and mitosis in the rapamycin‐dependent proteome, possibly reflecting the anti‐proliferative effects of rapamycin (Zaragoza et al., 1998) and the role of mTOR in cell proliferation (Dowling et al., 2010).

Overall, we here provide an unbiased evaluation of rapamycin's specificity towards mTOR in mammalian cells in unprecedented depth. Given the immense potential that rapamycin and its analogues have as anti‐ageing compounds or in therapeutics, these findings provide important insight for both basic and translational research and aim at improving the applicability and specificity of its use in humans. Future studies using analogous mTOR^RR^ animal models (e.g., *Drosophila* or mice) that express rapamycin‐resistant mTOR from the endogenous locus will be important to assess whether rapamycin exhibits similar extraordinary selectivity and specificity towards mTOR also at the organismal level and in individual tissues.

## 4 METHODS

### 4.1 Cell culture

All cell lines were grown at 37°C, 5% CO_2_. Human female embryonic kidney HEK293FT cells (#R70007; Invitrogen; RRID: CVCL_6911) were cultured in high‐glucose Dulbecco's Modified Eagle Medium (DMEM) (#41965–039; Gibco) supplemented with 10% fetal bovine serum (FBS) (#F7524; Sigma, or #S1810; Biowest) and 1% Penicillin–Streptomycin (#15140–122; Gibco).

HEK293FT cells were purchased from Invitrogen. The identity of the HEK293FT cells was validated by the Multiplex human Cell Line Authentication test (Multiplexion GmbH), which uses a single nucleotide polymorphism (SNP) typing approach, and was performed as described at www.multiplexion.de. All parental and edited cell lines were regularly tested for *Mycoplasma* contamination using a PCR‐based approach and were confirmed to be *Mycoplasma*‐free.

### 4.2 Cell culture treatments

To allosterically inhibit mTOR, rapamycin (#S1039; Selleckchem), everolimus (#S1120; Selleckchem) or temsirolimus (# S1044; Selleckchem) were dissolved in DMSO and added directly into full cell culture media at a final concentration of 20 nM unless otherwise indicated in the figure legends. Treatments were performed for the times described in the figure legends. DMSO was used as a negative control for all treatments. Torin1 (#4247; Tocris) was used as an ATP‐competitive mTOR inhibitor and added in the culture media at a final concentration of 250 nM for 1 h.

Amino acid (AA) starvation experiments were performed as described previously (Demetriades et al., 2014; Tsokanos et al., 2016). In brief, custom‐made starvation media were formulated according to the Gibco recipe for high‐glucose DMEM specifically omitting all AAs. The media were filtered through a 0.22‐μm filter device and tested for proper pH and osmolarity before use. For the respective AA‐replete (+AA) treatment media, commercially available high‐glucose DMEM was used (#41965039; Thermo Fisher Scientific). All treatment media were supplemented with 10% dialyzed FBS (dFBS) and 1x Penicillin–Streptomycin (#15140–122; Gibco). For this purpose, FBS was dialysed against 1× PBS through 3500 MWCO dialysis tubing. For basal (+AA) conditions, the culture media were replaced with +AA treatment media 1 h before lysis. For amino‐acid starvation (−AA), culture media were replaced with starvation media for 1 h.

### 4.3 Antibodies

Antibodies against phospho‐p70 S6K (Thr389) (#9205), p70 S6K (#9202 for Figure 1c,e; #97596 for Figure 1d,f), FLAG (#2368) and mTOR (#2983) were purchased from Cell Signaling Technology. The anti‐human tubulin (#T9026) antibody was purchased from Sigma.

### 4.4 Plasmid DNA transfections

Plasmid DNA transfections in HEK293FT cells were performed using Effectene transfection reagent (#301425; Qiagen) according to the manufacturer's instructions.

### 4.5 Generation of the gene‐edited mTOR^RR^ cell line

The rapamycin‐resistant mTOR (mTOR^RR^) HEK293FT cell line was generated by gene‐editing using the pX459‐based CRISPR/Cas9 system (Ran et al., 2013). The Ser2035Thr amino acid substitution was introduced by homology‐directed repair (HDR) using a single‐stranded DNA oligo as donor template (Paquet et al., 2016). The respective nucleotide change in the MTOR cDNA (NM_004958.4) is c.6103 T > A (Figure S1b). To target the *MTOR* gene locus, a sgRNA expression vector was generated by cloning appropriate DNA oligonucleotides (Table S8) in the BbsI restriction sites of pX459 (#62988; Addgene). The resulting plasmid was co‐transfected together with 1 μL of a donor DNA oligo (100 μM) containing the Ser2035Thr (TCT > ACT) substitution (Table S8) in HEK293FT cells. Transfected cells were selected with 3 μg/mL puromycin (#A11138‐03; Thermo Fisher Scientific) 36–48 h post‐transfection. Single‐cell clones were generated by FACS sorting and individual mutant clones were validated by genomic DNA sequencing and functional assays to assess the responsiveness of mTORC1 to rapamycin (and other rapalogs).

### 4.6 Gene expression analysis (RNA‐seq)

To analyze gene expression changes via RNA‐seq experiments, total mRNA was isolated using QIAshredder columns (#79656; Qiagen) and the RNeasy Plus Mini Kit (#74034; Qiagen) according to the manufacturer's instructions. RNA‐seq experiments were performed by the Max Planck Genome Centre (MPGC) Cologne, Germany (https://mpgc.mpipz.mpg.de/home/). RNA quality was assessed with an Agilent Bioanalyzer (Nanochip). Library preparation was done according to NEBNext Ultra™ II Directional RNA Library Prep Kit for Illumina (#E7760L; New England Biolabs) including polyA enrichment and addition of ERCC RNA spike‐ins. Libraries were quality controlled by Agilent TapeStation or LabChip GX or GX Touch (PerkinElmer). Sequencing‐by‐synthesis was performed on a HiSeq 3000 (Illumina) with single read mode 1 × 150 bp. Data from one representative RNA‐seq experiment, out of two independent replicates, are shown in this manuscript. Each experiment was performed from 3 independent biological replicates. The raw data from both RNA‐seq experiments are available in the NCBI Sequence Read Archive (see also the *Data Availability Statement* section).

### 4.7 Cell lysis and immunoblotting

For standard SDS‐PAGE and immunoblotting experiments, cells from a well of a 12‐well plate were treated as indicated in the figures and lysed in 250 μL of ice‐cold Triton lysis buffer (50 mM Tris pH 7.5, 1% Triton X‐100, 150 mM NaCl, 50 mM NaF, 2 mM Na‐vanadate, 0.011 gr/mL beta‐glycerophosphate) supplemented with 1x PhosSTOP phosphatase inhibitors (#4906837001; Roche) and 1× complete protease inhibitors (#11836153001; Roche) for 10 min on ice. Samples were clarified by centrifugation (~22,000 *g*, 10 min, 4°C) and supernatants were boiled in 1x SDS sample buffer (5× SDS sample buffer: 350 mM Tris–HCl pH 6.8, 30% glycerol, 600 mM DTT, 12.8% SDS, 0.12% bromophenol blue). Protein samples were subjected to electrophoretic separation on SDS‐PAGE and analysed by standard Western blotting techniques. In brief, proteins were transferred to nitrocellulose membranes (#10600002 or #10600001; Amersham) and stained with 0.2% Ponceau solution (#33427‐01; Serva) to confirm equal loading. Membranes were blocked with 5% skim milk powder (#42590; Serva) in PBS‐T [1× PBS, 0.1% Tween‐20 (#A1389; AppliChem)] for 1 h at room temperature, washed three times for 10 min with PBS‐T and incubated with primary antibodies [1:1000 in PBS‐T, 5% bovine serum albumin (BSA; #10735086001; Roche)] rotating overnight at 4°C. The next day, membranes were washed three times for 10 min with PBS‐T and incubated with appropriate HRP‐conjugated secondary antibodies (1:10000 in PBS‐T, 5% milk) for 1 h at room temperature. Signals were detected by enhanced chemiluminescence (ECL) using the ECL Western Blotting Substrate (#W1015; Promega) or SuperSignal West Pico PLUS (#34577; Thermo Scientific) and SuperSignal West Femto Substrate (#34095; Thermo Scientific) for weaker signals. Immunoblot images were captured on films (#28906835; GE Healthcare, #4741019289; Fujifilm).

### 4.8 Co‐immunoprecipitation (co‐IP)

For co‐IP experiments, 1.5 × 10^6^ cells were transiently transfected with the indicated plasmids and lysed 36 h post‐transfection in IP lysis buffer (50 mM Tris pH 7.5, 0.3% CHAPS, 150 mM NaCl, 50 mM NaF, 2 mM Na‐vanadate, 0.011 g/mL β‐glycerophosphate, 1× PhosSTOP phosphatase inhibitors and 1× complete protease inhibitors). FLAG‐tagged proteins were incubated with 30 μL pre‐washed anti‐FLAG M2 affinity gel (Sigma; #A2220) for 3 h at 4°C and washed four times with IP wash buffer (50 mM Tris pH 7.5, 0.3% CHAPS, 150 mM NaCl and 50 mM NaF). Samples were then boiled for 6 min in 1× SDS sample buffer and analysed by immunoblotting using appropriate antibodies.

### 4.9 Quantitative whole‐cell proteomics

For mass‐spectrometry experiments, HEK293FT cells were cultured in 10 cm dishes in 10 mL of complete culture media as described above. Rapamycin (or DMSO as negative control) were added directly in the culture media for 24 or 48 h. Experiments were performed with 5 independent biological replicates per condition (1 × 10 cm dish per replicate). In brief, cells were scraped, collected in 1.5 mL tubes on ice, washed in serum‐free media, pelleted by centrifugation (500 *g*, 3 min), and cell pellets were snap‐frozen in liquid nitrogen and stored at −80°C.

#### 4.9.1 Sample preparation

For sample preparation for quantitative proteomic analysis, cell pellets were lysed in 6 M guanidinium chloride (GdmCl) supplemented with 2.5 mM TCEP (tris(2‐carboxyethyl)phosphine), 10 mM CAA (chloroacetamide) and 100 mM Tris–HCl at room temperature. Samples were boiled at 95°C for 10 min and sonicated for 30 s for 10 cycles, with 30 s breaks on high‐performance mode with Bioruptor Plus (#B01020001; Diagenode). Samples were centrifuged at 20,000 *g* for 20 min at RT, supernatants were diluted 10 times with 20 mM Tris and protein concentration was measured using Nanodrop 2000 (#ND‐2000; Thermo Fischer Scientific). Three hundred micrograms of each sample were diluted 10 times with 20 mM Tris and digested with 1.5 μL of Mass Spectrometry Grade Trypsin Gold (#V5280; Promega) at 37°C overnight. Digestion was stopped by adding 50% FA to the reaction at a final concentration of 1%. Samples were centrifuged at 20,000 *g* for 10 min at RT and supernatants were collected. C‐18‐SD StageTips were washed and equilibrated sequentially with 200 μL methanol, 200 μL 40% ACN (acetonitrile)/0.1% FA (formic acid) and 200 μL 0.1% FA by centrifugation, each step for 1 min at RT. Samples were diluted with 0.1% FA, loaded in StageTips and centrifuged for 1–2 min at RT. StageTips were then washed twice with 200 μL 0.1% FA. Tryptic peptides were eluted from StageTips with 100 μL 40% acetonitrile (ACN)/0.1% formic acid (FA) by centrifugation (300 *g*, 4 min, RT). Eluates were dried in a Speed‐Vac at 45°C for 40–45 min and resuspended in 20 μL 0.1% FA. Four micrograms of the peptides were dried in a Speed‐Vac and stored at −20°C.

#### 4.9.2 TMT10plex labelling

The dried tryptic peptides were reconstituted in 9 μL of 0.1 M TEAB (triethylammonium bicarbonate). Tandem mass tag (TMT10plex, #90110; Thermo Fisher Scientific) labelling was carried out according to the manufacturer's instructions with the following changes: 0.8 mg of TMT10plex reagent was re‐suspended with 70 μL anhydrous ACN. Seven microliters of TMT10plex reagent in ACN were added to 9 μL of clean peptide in 0.1 M TEAB. The final ACN concentration was 43.75% and the ratio of peptides to TMT10plex reagent was 1:20. After 60 min incubation, the reaction was quenched with 2 μL 5% hydroxylamine. Labelled peptides were pooled, dried, re‐suspended in 200 μL 0.1% FA, split in two equal parts and desalted using home‐made STAGE tips (Li et al., 2021).

#### 4.9.3 Fractionation of TMT10plex‐labelled peptide mixture

One of the two parts was fractionated on a 1 mm × 150 mm ACQUITY column, packed with 130 Å, 1.7 μm C18 particles (#186006935; Waters) using an Ultimate 3000 UHPLC (Thermo Fisher Scientific). Peptides were separated at a flow of 30 μL/min with an 88 min segmented gradient from 1% to 50% buffer B for 85 min and from 50% to 95% buffer B for 3 min; buffer A was 5% ACN, 10 mM ammonium bicarbonate, buffer B was 80% ACN, 10 mM ammonium bicarbonate. Fractions were collected every 3 min, pooled in two passes (fraction 1 + 17, fraction 2 + 18, …, etc.) and dried in a vacuum centrifuge (Eppendorf).

#### 4.9.4 LC–MS/MS analysis

Dried fractions were re‐suspended in 0.1% FA, separated on a 50 cm, 75 μm Acclaim PepMap column (#164942; Thermo Fisher Scientific) and analysed on a Orbitrap Lumos Tribrid mass spectrometer (Thermo Fisher Scientific) equipped with a FAIMS device (Thermo Fisher Scientific). The FAIMS device was operated in two compensation voltages, −50 V and −70 V. Synchronous precursor selection based MS3 was used for the acquisition of the TMT10plex reporter ion signals. Peptide separations were performed on an EASY‐nLC1200 using a 90 min linear gradient from 6% to 31% buffer; buffer A was 0.1% FA, buffer B was 0.1% FA, 80% ACN. The analytical column was operated at 50°C. Raw files were split based on the FAIMS compensation voltage using FreeStyle (Thermo Fisher Scientific).

#### 4.9.5 Data analysis

Proteomics data was analysed using MaxQuant, version 1.6.17.0 (Cox & Mann, 2008). The isotope purity correction factors, provided by the manufacturer, were included in the analysis. Differential expression analysis was performed using limma, version 3.34.9 (Ritchie et al., 2015) in R, version 3.4.3 (R Core Team, 2017).

### 4.10 Gene ontology analysis and data presentation

Gene Ontology (GO) term enrichment analysis was performed using the Database for Annotation, Visualization and Integrated Discovery (DAVID) tool (Huang da et al., 2009a, 2009b) of the Flaski toolbox (Iqbal et al., 2021) (https://flaski.age.mpg.de, developed and provided by the MPI‐AGE Bioinformatics core facility). For the RNA‐seq experiments, either all significantly changing genes (adjusted *p*‐value <0.05) or selected genes whose expression levels change significantly between rapamycin‐ and DMSO‐treated cells with Log_2_‐transformed fold change (Log_2_FC) values lower than −0.75 (downregulated by rapamycin treatment) or higher than +0.8 (upregulated by rapamycin treatment), roughly corresponding to the top and bottom 5% of the dataset, were used for the DAVID GO analysis (for GOTERM_CC_FAT, GOTERM_BP_FAT). The selection criteria for each analysis are described in the respective figure legends.

For the DAVID GO analysis of quantitative proteomics experiments, either all significantly changing proteins (adjusted *p*‐value <0.05) or selected proteins whose intensity changes significantly between rapamycin‐ and DMSO‐treated cells with Log_2_FC < −0.55 and Log_2_FC > +0.4 (24 h treatments) or Log_2_FC < −0.65 and Log_2_FC > +0.45 (48 h treatments) were used (for GOTERM_CC_FAT, GOTERM_BP_FAT). Cut‐offs were selected based on the distribution of the ranked Log_2_FC values in each dataset to include only proteins whose levels change robustly in response to rapamycin treatment in control cells. The human proteome was used as reference list for all analyses.

Cell plots were generated using the DAVID and Cell plot apps in Flaski and include the 15 most significant GO terms from each analysis. The full list of genes/proteins that were detected in each experiment was used for generating the Volcano plots. The respective graphs were prepared using the Scatter plot app in Flaski and labelled in Adobe Photoshop (v. 23.4.2). Significantly changing genes/proteins are represented by blue (downregulated by rapamycin) or red (upregulated by rapamycin) dots. Proteins/genes whose levels change strongly upon rapamycin treatment (based on the selection criteria described above for each experiment) are shown as blue or red dots with black outline.

Heatmaps were generated using the homonymous app in Flaski, and hierarchical clustering was performed using Euclidean distance and Ward's method (Iqbal et al., 2021).

## AUTHOR CONTRIBUTIONS

Experimental work: FA, NG; data analysis: FA, CD; project design & conceptualization: CD; project supervision: CD; funding acquisition: CD; figure preparation: FA, NG, CD; manuscript draft: FA, CD. All authors approved the final version of the manuscript and agree on the content and conclusions.

## CONFLICT OF INTEREST STATEMENT

The authors declare no competing interests.

## CODE AVAILABILITY

No code was generated in this study.

## Supporting information


Figures S1–S4

Table S1

Table S2

Table S3

Table S4

Table S5

Table S6

Table S7

Table S8

## Data Availability

The data that support the findings of this study (uncropped immunoblots) or any additional information required to reanalyse the data reported in this paper are available from the corresponding author upon reasonable request. Other datasets that are produced in this study are available in the following databases:
RNA‐seq data: NCBI Sequence Read Archive (SRA) PRJNA872474 (www.ncbi.nlm.nih.gov/sra/PRJNA872474). Mass spectrometry proteomics: PRIDE (Perez‐Riverol et al., 2022) PXD038051 (www.ebi.ac.uk/pride/archive/projects/PXD038051).
