# Supplementary material for: Unbiased evaluation of rapamycin's specificity as an mTOR inhibitor
Source: Aging Cell. 2023 May 24;22(8):e13888. doi: 10.1111/acel.13888 (PMC10410055; doi:10.1111/acel.13888)
Supplement: Supplementary file 1 — Figures S1–S4 [file ACEL-22-e13888-s003.pdf]

Supplementary Information

Figures S1-S4 and Tables S1-S8

Supplementary Tables

Table S1. Differential gene expression analysis in control (WT) and $mTOR^{RR}$ HEK293FT cells upon rapamycin treatment.

Table S2. List of genes used for the GO analysis and associated GO terms from the RNA-seq analysis in Rapa- vs DMSO-treated WT cells (only strongly affected genes).

Table S3. List of genes used for the GO analysis and associated GO terms from the RNA-seq analysis in Rapa- vs DMSO-treated WT cells (all significantly changing genes).

Table S4. Differential protein expression analysis in control (WT) and $mTOR^{RR}$ HEK293FT cells upon rapamycin treatment.

Table S5. List of proteins used for the GO analysis and associated GO terms from the proteomic analysis in 24 h Rapa- vs DMSO-treated WT cells (all significantly changing proteins).

Table S6. List of proteins used for the GO analysis and associated GO terms from the proteomic analysis in 48 h Rapa- vs DMSO-treated WT cells (all significantly changing proteins).

Table S7. List of proteins used for the GO analysis and associated GO terms from the proteomic analysis in 24 h Rapa- vs DMSO-treated WT cells (only strongly affected proteins).

Table S8. List of DNA oligonucleotides used in this study.

Supplementary Figures

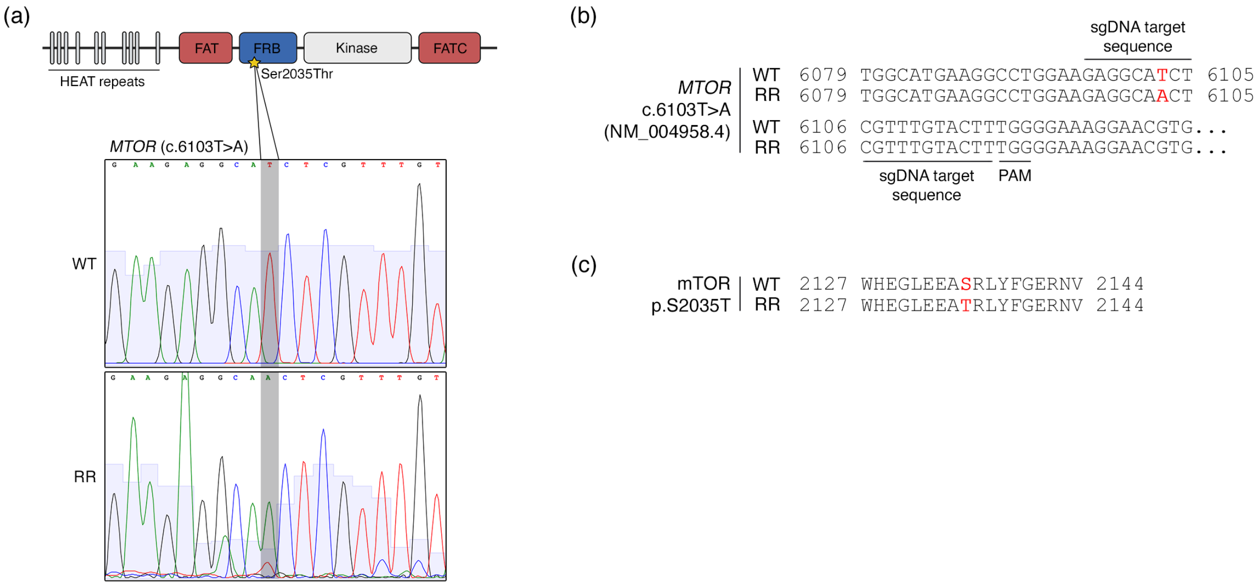

Figure S1. Characterization of the *MTOR* genomic alterations in the mTOR^{RR} HEK293FT cells. (a-c) CRISPR/Cas9-mediated gene-editing of *MTOR*. The associated genomic changes in *MTOR* were validated by Sanger sequencing (a). The resulting changes in the mTOR cDNA (c.6103T>A) and protein sequence (p.S2035T) are shown in (b) and (c), respectively. The sgDNA target sequence and PAM site are marked on the *MTOR* sequence in (b). The position of the modified nucleotide / amino acid residue is shown in red.

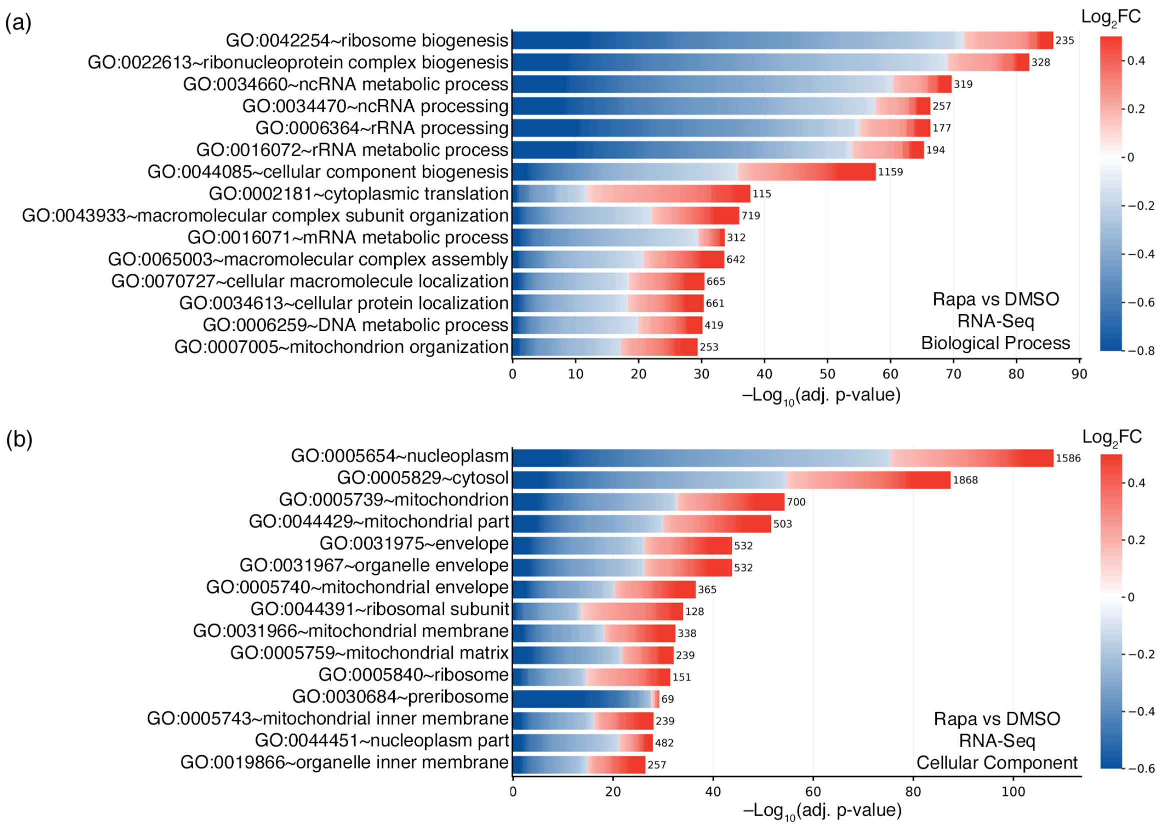

Figure S2. GO analysis of the rapamycin-induced changes in the transcriptome of WT HEK293FT cells.

(a) Biological process (BP) GO term analysis using all genes that are significantly (adj. p-value < 0.05) down- (blue) or upregulated (red) by rapamycin in WT cells, as described in Fig. 2b. The color of each box in the cell plot represents log-transformed fold change values for each gene in rapamycin- vs DMSO-treated cells. The number of genes in the selected dataset for each GO term is shown on the right side of each bar.

(b) As in (a), but for Cellular Component (CC) GO term analysis.

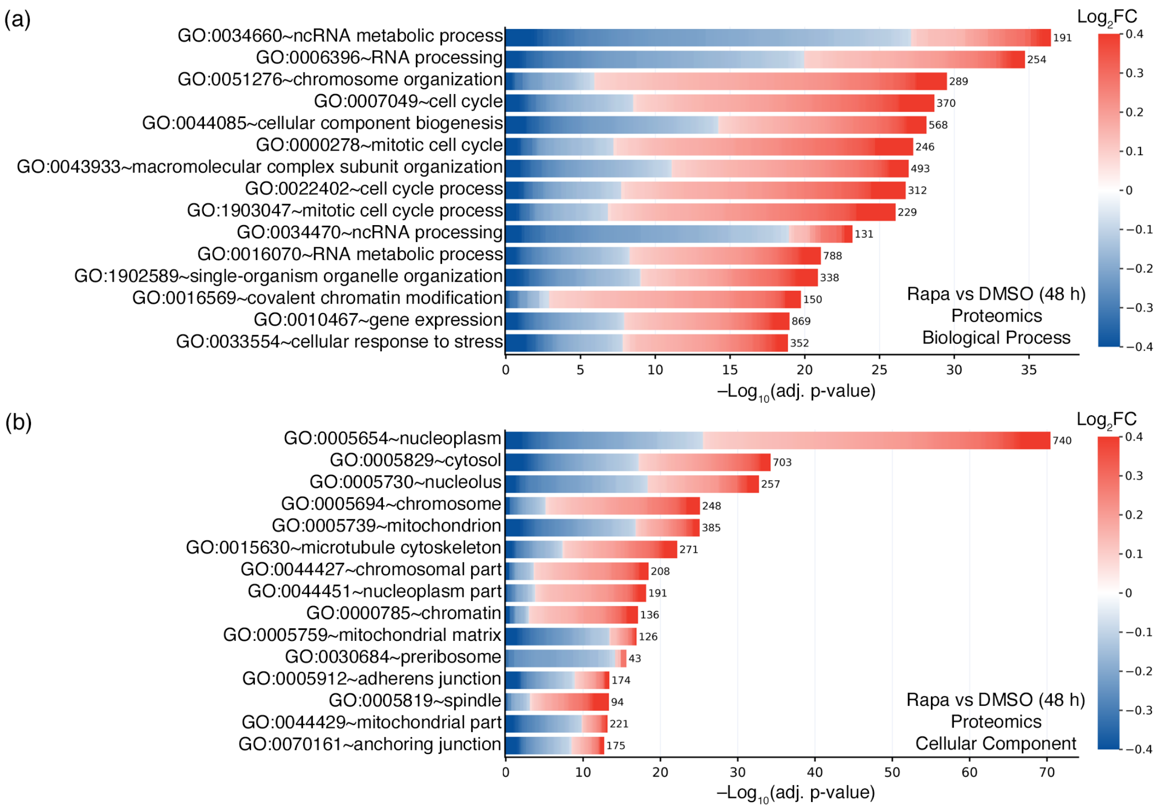

Figure S3. GO analysis of the rapamycin-induced changes (48 h) in the proteome of WT HEK293FT cells.

(a) Biological process (BP) GO term analysis using all proteins that are significantly down- (blue) or upregulated (red) by rapamycin (48 h) in WT cells, as described in Fig. 3c. The color of each box in the cell plot represents log-transformed fold change values for each protein in rapamycin- vs DMSO-treated cells. The number of proteins in the selected dataset for each GO term is shown on the right side of each bar.

(b) As in (a), but for Cellular Component (CC) GO term analysis.

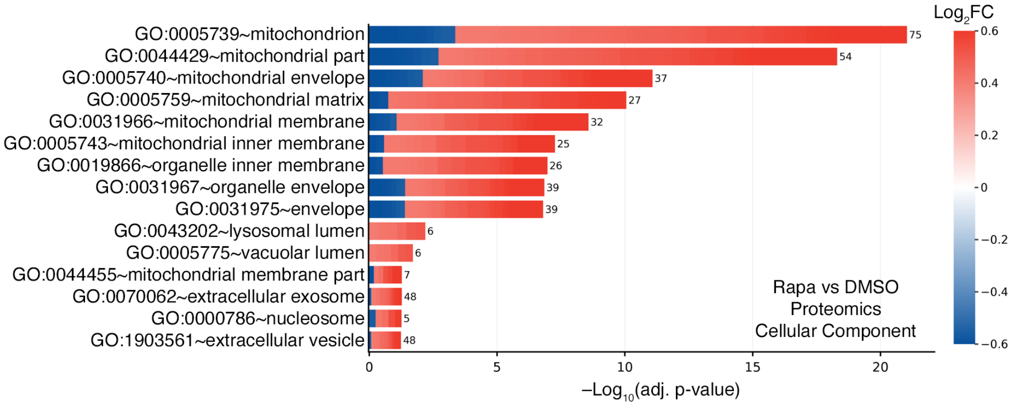

Figure S4. GO analysis of the robust rapamycin-induced changes (24 h) in the proteome of WT HEK293FT cells.

Cellular Component (CC) GO term analysis using only the proteins that are strongly down- (blue) or upregulated (red) by rapamycin (24 h) in WT cells, as described in Fig. 3b. The color of each box in the cell plot represents log-transformed fold change values for each protein in rapamycin- vs DMSO-treated cells. The number of proteins in the selected dataset for each GO term is shown on the right side of each bar.
